# Characterization of a heat responsive UDP: Flavonoid glucosyltransferase gene in tea plant (*Camellia sinensis*)

**DOI:** 10.1371/journal.pone.0207212

**Published:** 2018-11-26

**Authors:** Xiaojia Su, Wenzhao Wang, Tao Xia, Liping Gao, Guoan Shen, Yongzhen Pang

**Affiliations:** 1 Institute of Animal Sciences of Chinese Academy of Agricultural Sciences, Beijing, China; 2 Institute of Botany, the Chinese Academy of Sciences, Beijing, China; 3 School of Life Science, Anhui Agricultural University, Hefei, China; 4 The Institute of Medicinal Plant Development, Beijing, China; ICAR - National Research Center on Plant Biotechnology, INDIA

## Abstract

Tea plant (*Camellia sinensis*) accumulates abundant flavonoid glycosides that are the major bioactive ingredients in tea. Biosynthesis of flavonoid glycosides are catalyzed by UDP-glucosyltransferases (UGTs) that are widely present in plants. Among one hundred and seventy-eight *UGTs* genes that we have previously identified in tea plant, few of them have been functionally characterized. In the present study, we further identified *UGT73A17* gene that is responsible for the biosynthesis of a broad range of flavonoid glycosides. Sequence analysis revealed that the deduced UGT73A17 protein showed high identity with 7-*O*-glycosyltransferases at amino acid level and it was clustered into the clade containing several 7-*O*-glycosyltransferases from other plant species. Enzymatic assays revealed that the recombinant UGT73A17 protein (rUGT73A17) exhibited activity toward flavonols (kaempferol, quercetin, and myricetin), flavones (apigenin, luteolin, and tricetin), flavanone (naringenin), isoflavones (genistein) and epicatechin gallate, yielding 7-*O*-glucosides as the major *in vitro* products. In particular, rUGT73A17 displayed higher activity at high temperatures (eg. 50°C) than at low temperatures, which was consistent with its relatively high expression level at high temperatures. Two amino acid substitutions at I296L and V466A improved the enzymatic activity of rUGT73A17. Our study demonstrated that UGT73A17 is responsible for the biosynthesis of a broad range of flavonoid glucosides, which is also involved in heat response and quality of tea plant.

## Introduction

Tea is a fascinating and complex beverage that contains many bioactive ingredients including flavonoids. Among them, catechins (or flavan-3-ols), flavonols, and condensed tannins are the major components with pivotal bioactivities in tea. Catechins including epicatechin/catechin, epigallocaetchin/gallocatechin, epicatechin-3-gallate/catechin-3-gallate, and epigallocatechin-3-gallate/ gallocatechin-3-gallate are abundant in tea [1, 2]. Flavonols, including quercetin, kaempferol, myricitin and their glycosides (mono-, di-, and tri-), make up about 0.5–2.5% (w/w) in tea infusions. By contrast, apigenin, luteolin and their *C*-glycosides have been detected in tea with a very small fraction [1, 2].

Tea flavonoids are closely related to its typical flavour and to its pharmaceutical benefits on human health. Flavonol 3-*O*-glycosides have been found to induce velvety, mouth-drying, and mouth-coating sensations [3], while galloylated catechins have been found to confer astringent and bitter taste of tea [3–5]. In particular, tea flavonoids have been found to have numerous beneficial effects on human health, including the prevention of cancer and heart disease [6–9].

Tea plants are widely distributed in Asia, where they grow under various climate with extreme temperatures as high as 40–45°C. Elevated temperatures are known to induce an array of physiological and biochemical changes in plants, which greatly affect plant performance and further survival [10, 11, 12]. To deal with heat stress, plants resort to several mechanisms: activation of heat shock factors (HSFs) and heat shock proteins (HSPs) [10]; production of antioxidants, e.g. regulating the biosynthesis of flavonoids with a photoprotective and antioxidant capacity [13, 14].

Flavonoids are believed to be one of the major compounds that play major roles in plant response to various environmental cues, especially biotic and abiotic stresses by inhibiting the formation of ROS [15]. This may explain the different accumulation profiles of flavonoids under the diverse biotic and abiotic stress in plants [16]. In grapevine, MeJA up-regulated the expression of *CHS* and *UFGT* genes and resulted in a strong increase in anthocyanin contents [17]. Extreme temperature also affected the composition or concentration of flavonoids in plants. Low temperature has been shown to induce anthocyanin synthesis in various plant species [18]. Under high temperature, anthocyanin accumulation was decreased, which may result from accelerated anthocyanin degradation and inhibition of the expression of anthocyanin biosynthetic genes [19].

Flavonoids with one or more sugar units in different positions can increase their solubility and affect their biological activity [20]. The process of sugars attachment to flavonoids is defined as glycosylation, which is catalyzed by UDP-glycosyltransferases (UGTs). In a previous study, we have identified nearly two hundred *UGT* genes in tea plant by transcriptome analysis [21]. Among them, we found UGT84A22 exhibited catalytic activity toward phenolic acids, in particular gallic acid, to produce *β*-glucogallin. Meanwhile, UGT78A14 and UGT78A15 were responsible for the biosynthesis of flavonol 3-*O*-glucosides and flavonol 3- *O*-galactosides [21]. In an effort to functionally characterize additional *UGT* genes that are potentially involved in flavonoid glycosylation, we further identified and functionally characterized *UGT73A17*. We found rUGT73A17 protein exhibited glycosylation activity towards at least five classes of flavonoid aglycones, including flavonols, flavones, flavanones, isoflavones and epicatechin gallate. In particular, rUGT73A17 protein exhibited higher activity at high temperature than at low temperature in *in vitro* enzymatic assay. Expression analyses also confirmed that the expression level of *UGT73A17* was high under heat treatment. Our study demonstrated that UGT73A17 is active toward a broad range of flavonoid substrates with a regio-specificity at 7-*O* position, and *UGT73A17* is also involved in the heat response in tea plant.

## Materials and methods

### Cloning and expression of *UGT73A17* gene

The open reading frame of *UGT73A17* gene was obtained by searching the tea EST library as previously reported [22]. Its ORF was amplified with primer pair UGT73A17BF and UGT73A17SR (S1 Table) by using *pfu* polymerase and subcloned into the *Escherichia coli* expression vector pMAL-c2X (New England Biolabs, MA, USA). The resulting vector pMAL-c2X-UGT73A17 was sequenced for correct reading frame and then transformed into *E*. *coli* Novablue competent cells for recombinant protein expression. The MBP-fusion protein was induced and purified according to the manufacturer’s instruction with the addition of 0.5 mM isopropyl- *β*-D-thiogalactoside for induction.

### Plant materials, stress treatment and chemicals

Tea samples (*Camellia sinensis* cultivar Shuchazao) for qRT-PCR were all collected from the Horticultural Research Station of Anhui Agricultural University. The leaves for heat treatment were collected and then immediately put into the MS liquid medium at 50°C for 0.5 h, 1 h and 6 h, and then recovered to 20°C for 0.5 h and 1 h, and 20°C was used as control. The leaves were then rapidly frozen in liquid nitrogen and stored at -80°C until use.

Substrates tested in the present study were purchased from Tongtian Limited Co., Ltd (Shanghai, China) and Indofine (Hillsborough, NJ, USA). UDP-glucose was purchased from Sigma-Aldrich (Oakville, CA, USA). Chemicals used in this study were of analytical or HPLC grade.

### Sequence analyses

Multiple sequence alignment of the deduced amino acid sequences of UGTs were carried out by using DNAMAN software. Predicted amino acid sequences of UGTs were aligned using a Clustal W program, and then used for phylogenetic analysis. The neighbor-joining phylogenetic tree was constructed with 1000 bootstrap replicates using the software MEGA6.0 [23].

### Enzyme assays and kinetic analyses

The rUGT73A17 protein (3–6 μg) was incubated in a 50 μL reaction containing 100 mM Tris-HCl (pH = 7.0), 1 mM DTT, 0.5 mM substrates, and 4 mM UDP-glucose for 0.5 h. To test the optimum pH value, 100 mM Tris-HCl with pH value of 4.0, 5.0, 6.0, 7.0, 8.0, and 8.5 were tested. To test the optimum temperature, enzymatic reactions were carried out at temperatures of 0°C, 10°C, 20°C, 30°C, 40°C, 50°C, 55°C and 60°C. All reactions were stopped by the addition of the same volume of methanol, followed by analysis on HPLC after centrifugation at 12 000 rpm for 30 min. Enzyme kinetic analysis was carried out in a final volume of 50 μL with 3 μg purified proteins and 100 mM Tris-HCl (pH 7.0) at 30°C or 50°C for 0.5 hour. The concentration of substrates ranged from 0 to 400 μM.

Samples were run on an HPLC 1260 (Agilent) system with an Eclipse XDB-C18 reverse phase column (4.6 × 150 mm, particle size 5 μm). The separation conditions on HPLC were as followed: a linear (A: B) elution gradient from 95% solvent A (0.1% formic acid, v/v) to 70% solvent B (CH3CN) over a 30 min period with a flow rate of 1 mL·min^-1^. Reaction products were monitored at 254 nm or 280 nm on diode array detector. The kinetic parameters including *K*_*m*_ and *K*_*cat*_ were calculated with the Lineweaver-Burk method. All the enzymatic assay were performed in triplicates, and data are presented as mean ±SD.

### Expression analysis by qPCR

Total RNAs were isolated from roots, stems, developing leaves, buds, and heat-treated leaves by using Fruit-mate kit for RNA purification (Takara, DaLian, China) and RNAiso Plus (Takara, DaLian, China). The first-strand cDNA was synthesized using the PrimeScript RT (Takara, DaLian). Quantitative RT-PCR was performed in reaction containing cDNA template, 10 μL SYBR Green PCR Master Mix (Takara), and 200 mM of each gene-specific primer in a final volume of 20 μL. The PCR procedure were carried out on a CFX96 optical reaction module (Bio-Rad, USA) as followed: 95°C for 30 s, followed by 40 cycles at 95°C for 5 s, and 60°C for 30 s (58°C for 30 s for root samples). Primer pairs for *UGT73A17* and *CHS1* were listed in S1 Table. All the treated samples were normalized using mRNA of the reference gene *GAPDH* as an internal control. All the qPCR reactions were performed in triplicates, and data are presented as mean ±SD.

### Site-directed mutagenesis

Two step PCR method was used to amplify the ORF of *UGT73A17* with mutations. In detail, the sequenced plasmid pMAL-c2X-UGT73A17 was used as template to amplify the two fragments of *UGT73A17* separately. One fragment was obtained with primer pair UGT73A17BF and UGT73A17-I296L-R, and the other fragment was obtained with primer pair UGT73A17-I296L-F and UGT73A17SR. The resulting two fragments were used as templates in a single PCR to amplify ORF with primer pair UGT73A17BF and UGT73A17SR, which resulted in a mutation at 296 amino acid. The ORF was subsequently cloned into the pMAL-c2X vector, sequenced for correction and used for protein expression in *E*. *coli*. Vectors with another single substitution UGT73A17-V466A, and double substitution UGT73A17-1 (I296L and V466A) were generated by using the same strategy with corresponding primer pairs (S1 Table), respectively. The enzyme activity and enzymatic kinetic parameters were determined with the aforementioned enzyme reactions and HPLC method at 30°C or 50°C. All the enzymatic assay were performed in triplicates, and data are presented as mean ±SD.

## Results

### Identification and sequence analysis of *UGT73A17* in tea plant

In order to globally investigate the flavonoid biosynthetic pathway in tea plant, we generated a leaf EST library from tea plant in a previous study [22]. In this EST library, a full-length *UGT* gene, was found to be highly represented, which may play important role in flavonoid glycosylation. This UGT was further designated as UGT73A17 by the UGT nomenclature committee. The open reading frame of *UGT73A17* was 1422 bp in length and it encodes a protein of 473 amino acids.

**Fig 1:**
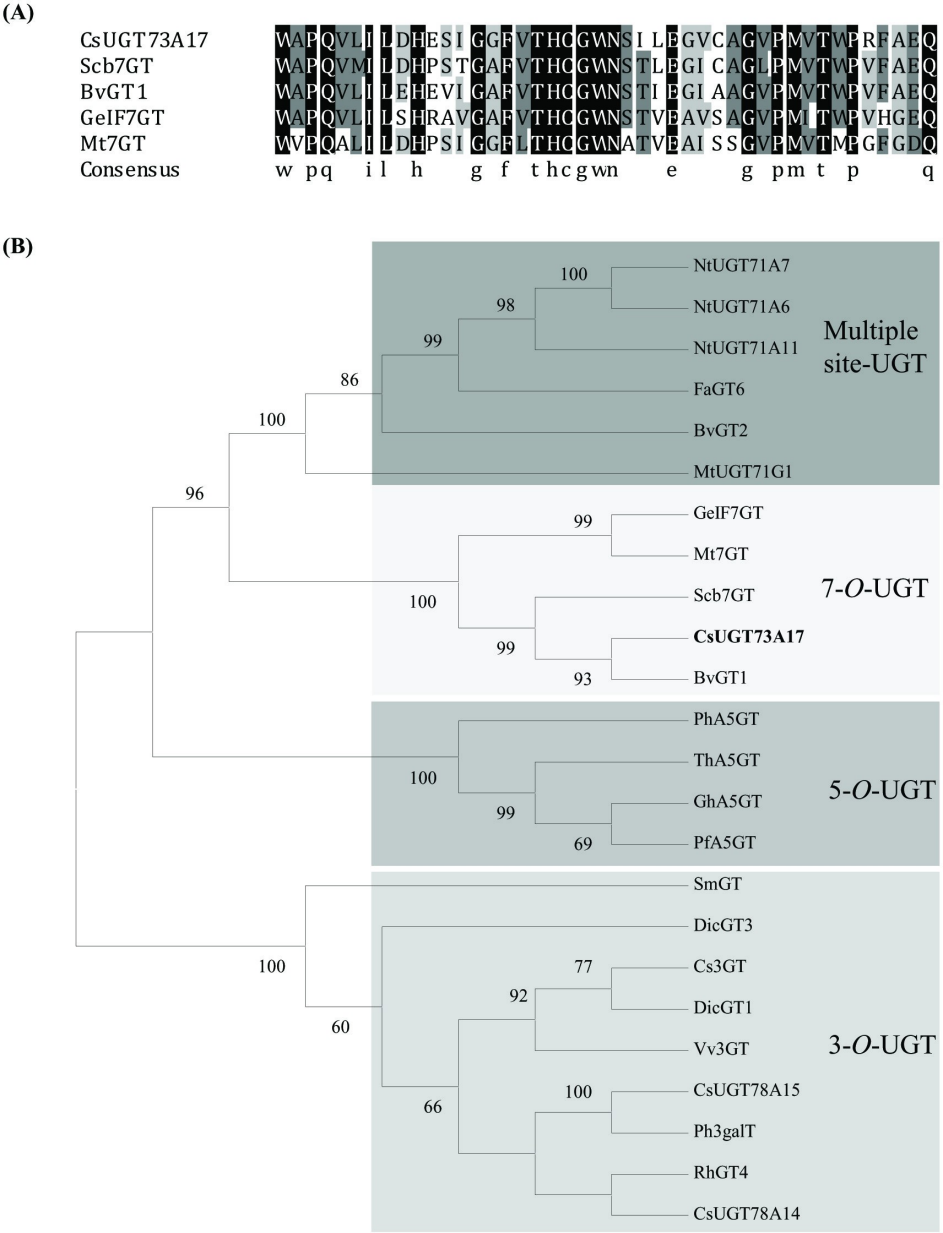
Sequence and phylogenetic analyses of the deduced UGT73A17 protein with other closely relative plant UGTs. (A) Multiple sequence alignment of the PSPG boxes of the deduced UGT73A17 protein with other functionally characterized UGTs at amino acid level. Identical amino acids were indicated in black background, amino acids with 75% and 50% similarities were indicated in dark gray and light gray, respectively. The GenBank accession numbers and plant species of other UGTs are: Scb7GT, BAA83484 (*Scutellaria baicalensis*); BvGT1, AAS94329 (*Beta vulgaris*); GeIF7GT, BAC78438 (*Glycyrrhiza echinata*) and Mt7GT, AAW56091 (*Medicago truncatula*); (B) Phylogenetic analyses of the deduced UGT73A17 protein with other functionally characterized plant UGTs. Protein sequences were aligned with Clustal W and a neighbor-joining tree was constructed using MEGA 6.0 software. Scale bar indicates the number of amino acid substitutions.

The deduced amino acid sequence of UGT73A17 showed 56%, 61%, 48%, and 41% identities with Scb7GT from *Scutellaria baicalensis*, BvGT1 from *Beta vulgaris*, GeIF7GT from *Glycyrrhiza echinata* and Mt7GT from *Medicago truncatula* (S1 Fig) [24–27]. In particular, UGT73A17 shared high identity within the conserved PSPG motif near its C-terminal domain (Fig 1A), and the last residue was glutamine (Q), which was believed to confer specificity for UDP-glucose as sugar donor [28].

Phylogenetic tree showed that UGT73A17 was clustered into a clade with four 7-*O*-UGTs (Scb7GT, BvGT1, GeIF7GT and Mt7GT) (Fig 1B). This clade was clearly separated from clades containing 3-*O*-UGT, 5-*O*-UGT and multiple-site-UGT in the phylogenetic tree (Fig 1B). This result implied that UGT73A17 was likely to be a 7-*O*-UGT.

### Expression of the rUGT73A17 protein in *E*. *coli*

In order to investigate the function of UGT73A17, it was successfully expressed in *E*. *coli* with an N-terminal maltose-binding protein tag (S2 Fig). The purified rUGT73A17 protein was tested with 17 flavonoid aglycones as potential substrates and UDP-glucose as sugar donor (S2 Table, S2 Fig).

**Fig 2:**
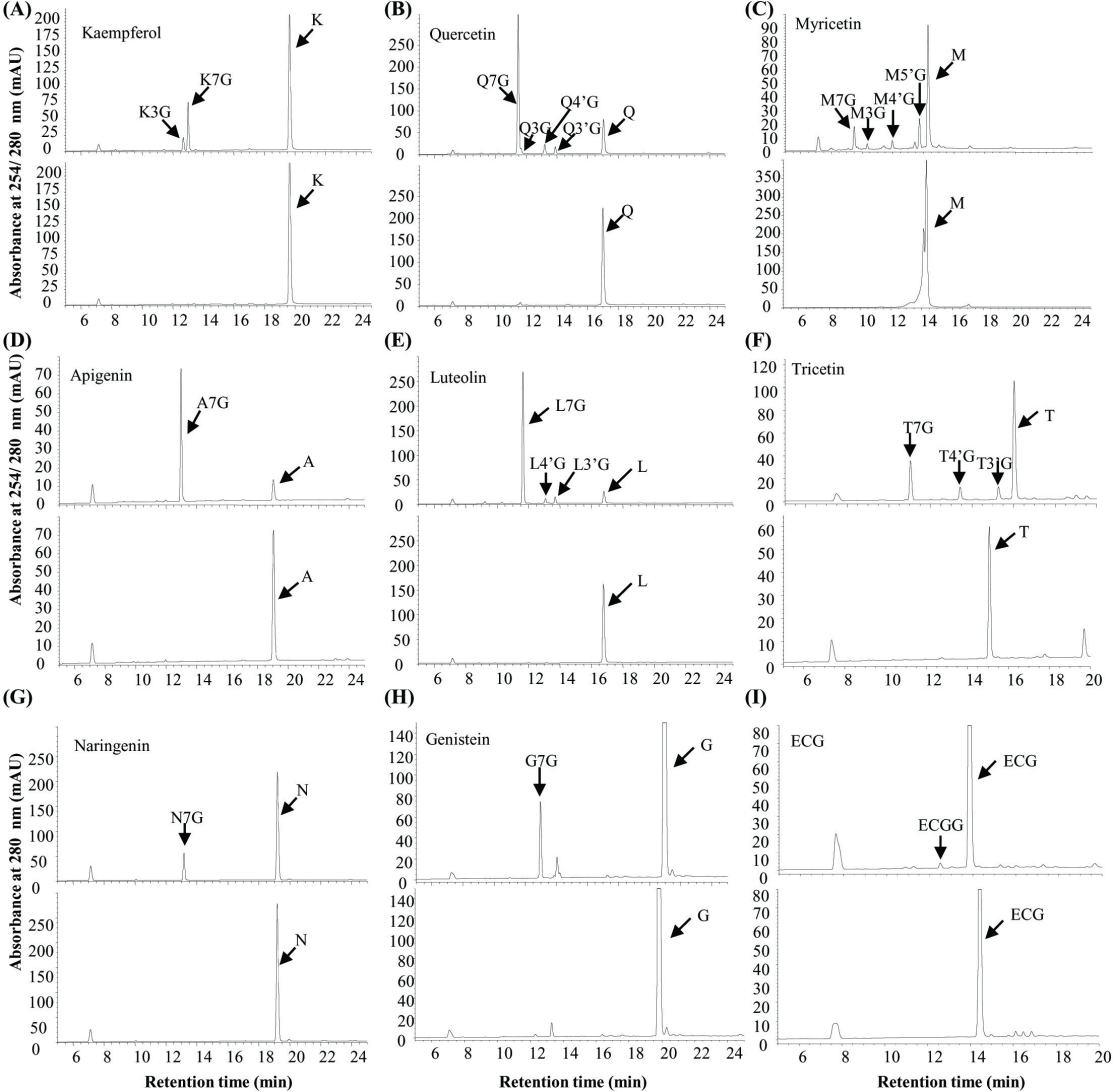
Analysis and identification of the enzymatic products of the recombinant UGT73A17 protein with various flavonoid substrates on HPLC. (A-C) Representative HPLC chromatographs of the enzymatic products of the recombinant UGT73A17 protein with kaempferol (A), quercetin (B) and myricetin (C) as substrates and UDP-glucose as sugar donor (upper panels). The reactions lacking the recombinant UGT73A17 protein were used as controls (lower panels); (D-I) Representative HPLC chromatograph of the enzymatic products of the recombinant UGT73A17 protein with apigenin (D), luteolin (E), tricetin (F), naringenin (G), genistein (H) and epicatechin gallate (I) as substrates and UDP-glucose as sugar donor (upper panels). The reactions lacking the recombinant UGT73A17 protein were used as controls (lower panels).

The rUGT73A17 protein exhibited activities toward flavonols (kaempferol, quercetin, and myricetin) and flavones (apigenin, luteolin, and tricetin) (Fig 2A–2F). The enzymatic products tested on HPLC showed that reaction with kaempferol as substrate produced K7G and K3G (Fig 2A). Four products (Q7G, Q3G, Q4'G, and Q3'G) were detected with quercetin as substrate, which was the same as for myricetin with four products (Fig 2B and 2C). The major products were compared with the authentic standards for all products with quercetin glucosides as representatives (S3 Fig). One product was detected for apigenin (A7G), three for luteolin (L7G, L4'G and L3'G) and three for tricetin (T7G, T4'G and T3'G) as substrates, respectively (Fig 2D–2F).

In addition, the rUGT73A17 protein also exhibited activity toward naringenin and genistein, and the enzymatic products were both 7-*O*-glucosides (Fig 2G and 2H). It is noted that in all the above reactions, the major products were 7-*O*-glucosides, which is consistent with the phylogenetic position of UGT73A17, which was grouped into the 7-*O*-UGT clade (Fig 1B). When catechins and their gallates were tested as potential substrates, only epicatechin gallate could be glucosylated (Fig 2I). The product had a m/z of 603 together with the aglycone fragment of m/z 441 (epicatechin gallate) by UPLC-MS/MS analysis (S4 Fig), indicating it was a glycosylation product of epicatechin gallate.

### Enzyme properties of the rUGT73A17 protein

**Fig 3:**
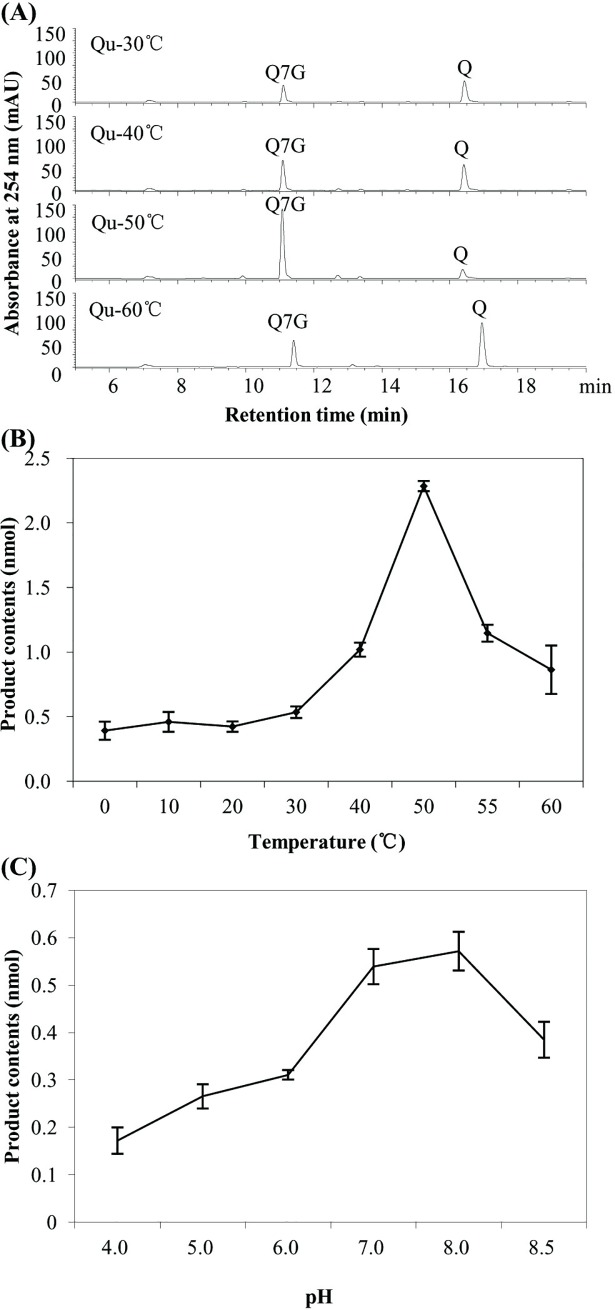
Enzyme property of the recombinant UGT73A17 protein toward quercetin at different temperatures and pH values. (A) Representative HPLC chromatograph of the enzymatic products of the recombinant UGT73A17 protein with quercetin as substrate at temperatures of 30°C, 40°C, 50°C and 60°C; (B) The effect of temperature on the activity of the recombinant UGT73A17 protein with quercetin as substrate; (C) The effect of pH values on the activity of the recombinant UGT73A17 protein with quercetin as substrate.

To better understand the properties of the rUGT73A17 protein, a gradient temperature and pH values were carried out in enzymatic reactions with quercetin as substrate. It revealed that the major product peak Q7G was less at 30°C and 40°C, but peaked at 50°C, followed by a decrease at 60°C (Fig 3A). When the temperature was lowered, the activity of rUGT73A17 protein at 0°C, 10°C and 20°C were lower than at 30°C (Fig 3B), and the product content was higher at 55°C than at 60°C (Fig 3B). Similar as quercetin, when apigenin and naringenin were tested, the products increased with temperature (S5A and S5B Fig). This result indicated the UGT73A17 protein was stable under high temperature condition, and it is likely involved in heat response in tea plant.

When various pH values were tested in the enzymatic reaction with quercetin as substrate, the activity of the rUGT73A17 protein increased with pH values ranging from 4.0 to 8.0, followed by a decrease at 8.5. This result indicated that the optimal pH value for UGT73A17 was basic with a pH value of around 8.0 (Fig 3C).

In addition, multiple glycosylated products were detected when reaction time was extended (S6A Fig, upper panel). At 2 h, more di-glucosides, even tri-glucosides could be detected as verified by UPLC-MS/MS (S6A Fig, lower panel, S6B–S6J Fig). This result indicated that the rUGT73A17 protein is capable of producing mono-, di-, tri-glucosides with extended reaction time.

### Kinetic parameters of the recombinant UGT73A17 protein

**Fig 4:**
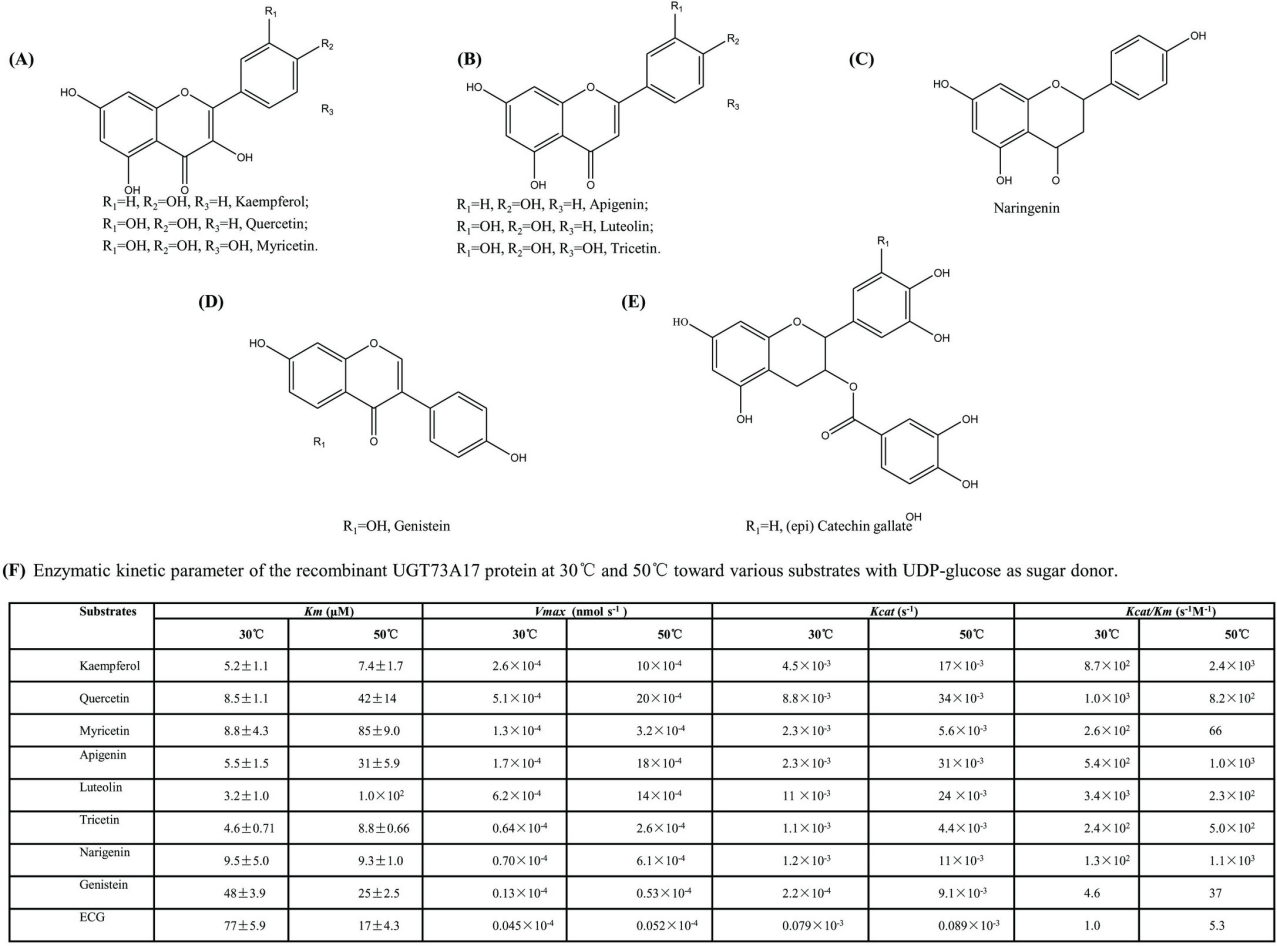
Flavonoid structures and enzymatic kinetic parameters of the recombinant UGT73A17 protein. (A-E) Structures of flavonols, flavones, naringein, genistin and epicatechin gallate that could be used as substrates by UGT73A17. (F) Enzymatic kinetic parameters of the recombinant UGT73A17 protein at 30°C and 50°C toward various substrates with UDP-glucose as sugar donor.

Since rUGT73A17 protein showed a higher activity at 50°C than at the commonly used temperature of 30°C, the enzymatic kinetic analyses were performed and compared under both 30°C and 50°C with nine substrates. It showed that the affinity of UGT73A17 toward kaempferol (*K*_*m*_ value of 5.2 μM at 30°C and 7.4 μM at 50°C) and luteolin (*K*_*m*_ value of 3.2 μM at 30°C and 1.0×10^2^ μM at 50°C) were relative higher than the other substrates (Fig 4F). The *V*_*max*_ of UGT73A17 with different substrates at 50°C were all higher than those at 30°C, indicating UGT73A17 is more active at 50°C than at 30°C. The values of *K*_*cat*_ with different substrates were about 1.1–13.4 fold higher at 50°C than at 30°C. The values of *K*_*cat*_/*K*_*m*_ for different substrates at 30°C and 50°C ranged from 5.3 for ECG to 2.4°C10^3^ for kaempferol (Fig 4F). In particular, the values of *K*_*cat*_/*K*_*m*_ for kaempferol, apigenin, tricetin, naringenin, genistein and ECG were about 2.0–8.5 fold higher at 50°C than at 30°C, indicating that the rUGT73A17 protein was more effective at 50°C than at 30°C. Taken together, the rUGT73A17 protein is more active at relative high temperature (eg. 50°C).

### Expression profile and heat stress response of *UGT73A17* gene

**Fig 5:**
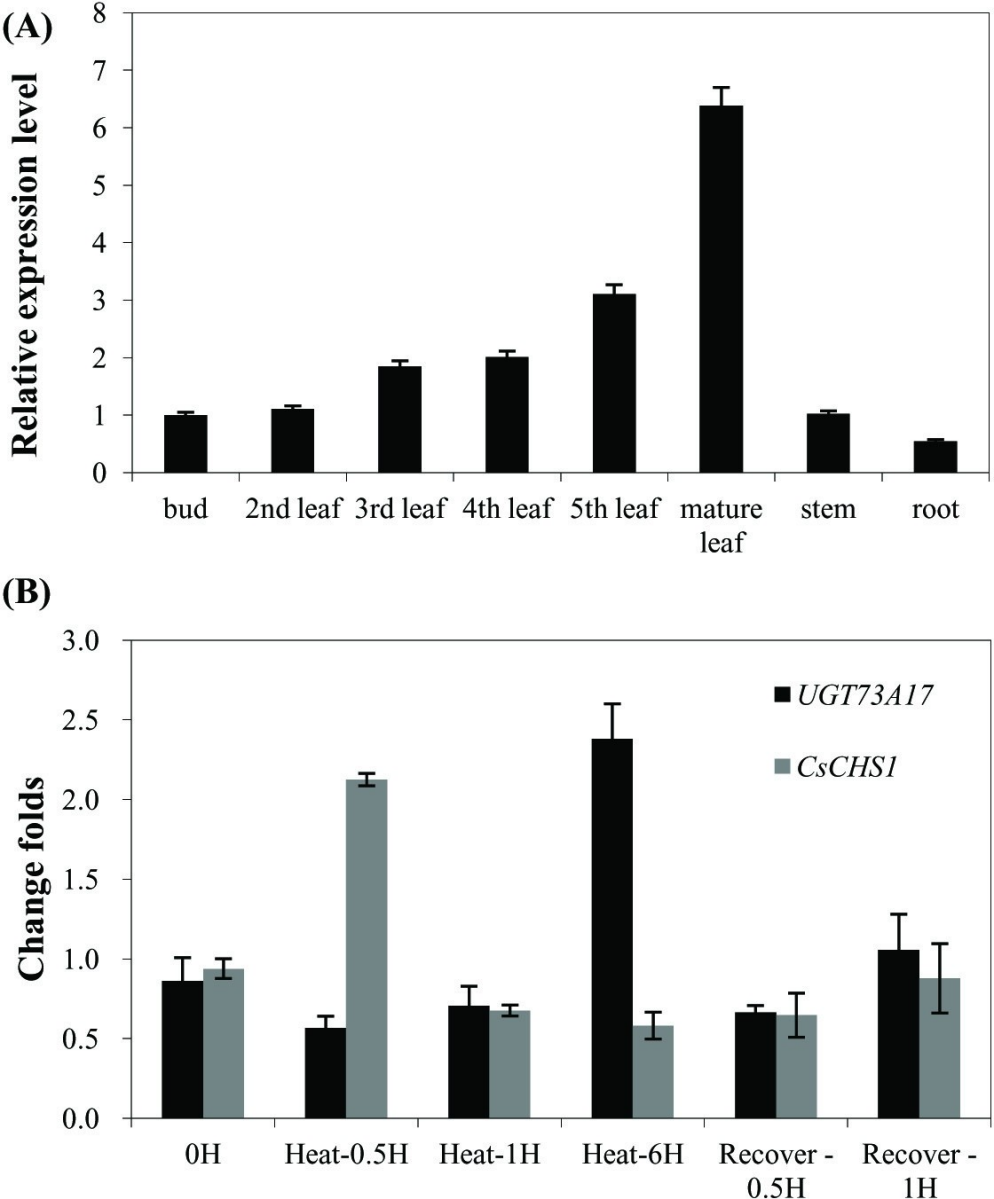
Expression analysis of *UGT73A17* gene in the tea plant. (A) The relative transcript level of *UGT73A17* gene in various tissues of the tea plant; (B) The relative transcript level of *UGT73A17* and *CsCHS1* genes in mature leaves under heat treatment at 50°C.

In order to investigate the expression profile of *UGT73A17* gene, its expression level was determined by qRT-PCR in various tissues. It revealed that *UGT73A17* was expressed in roots, stems, buds and leaves at different stages (Fig 5A). Relatively high expression levels were found in leaves, in particular mature leaves, which is more than 10 folds than in roots (Fig 5A).

Because *in vitro* enzymatic assays showed that UGT73A17 was more effective and active at 50°C, we thus measured the expression level of *UGT73A17* under 50°C in mature leaves in parallel with *CsCHS1* (*chalcone synthase*) gene. CHS catalyzes the first committed step in the flavonoid biosynthetic pathway and is essential in the inducible production of flavonoids in plant resistance [13]. It showed that the relative expression level of *CsCHS1* responded and increased quickly at 1 h, and *UGT73A17* responded at 6 h and increased by more than two folds (Fig 5B). After a recovery of 1 h, the expression level of *UGT73A17* dropped to the normal level (Fig 5B). This result indicated that *UGT73A17* is involved in the biosynthesis of flavonoid glycosides under heat treatment in tea leaves.

### Mutagenesis of rUGT73A17 protein

**Fig 6:**
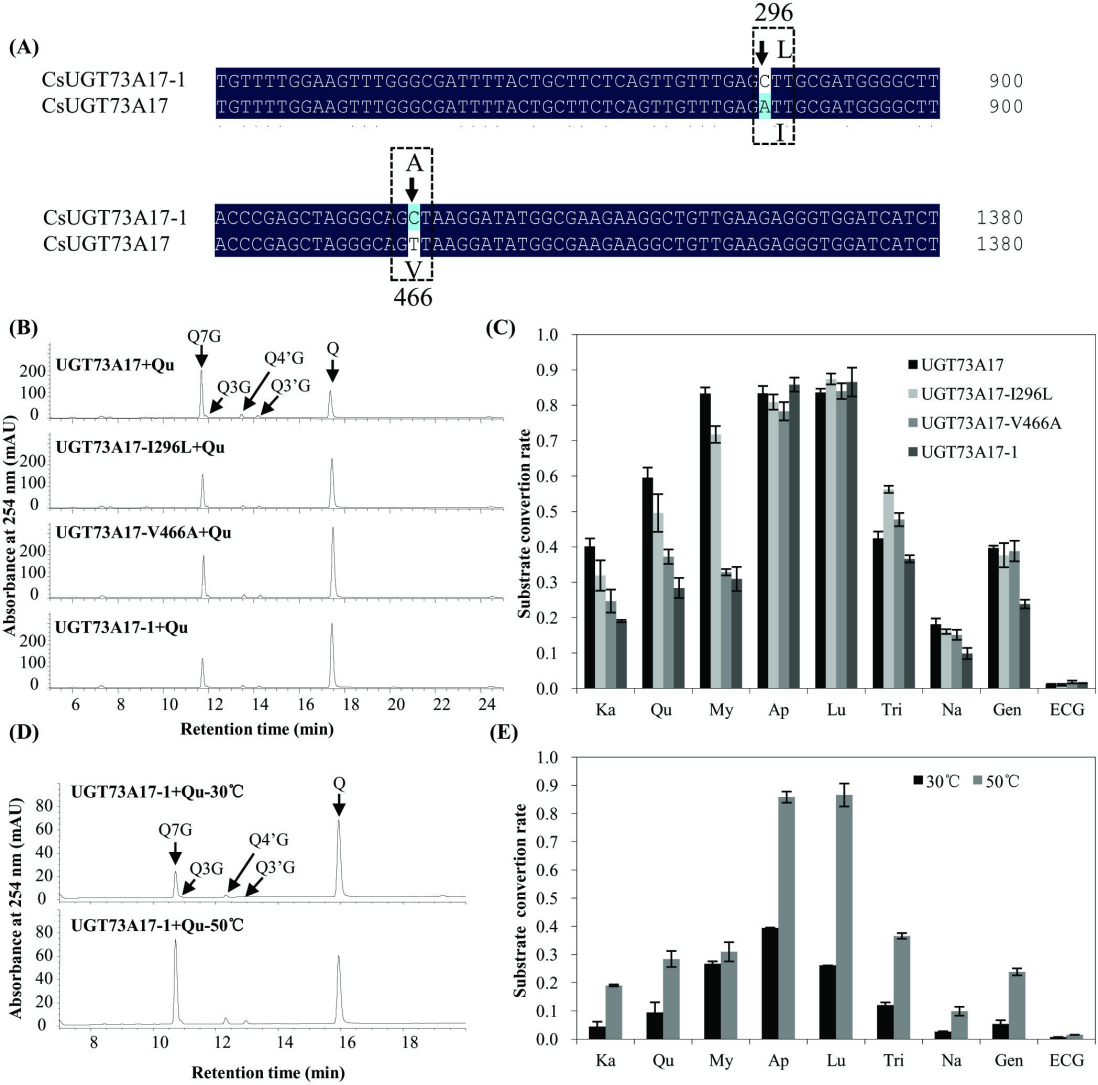
Analysis of the recombinant UGT73A17, UGT73A17-I296L, UGT73A17-V466A and UGT73A17-1 proteins. (A) Comparison of the nucleotide acid sequences of *UGT73A17* and *UGT73A17-1* genes. Different amino acids were indicated in dashed box; (B) Representative HPLC chromatograph of the enzymatic products of the recombinant UGT73A17, UGT73A17-I296L, UGT73A17-V466A and UGT73A17-1 proteins, with quercetin as substrate and UDP-glucose as sugar donor; Q, quercetin; (C) Comparison of the substrate conversion rate of the recombinant UGT73A17, UGT73A17-I296L, UGT73A17-V466A and UGT73A17-1, with nine flavonoid aglycones as substrates and UDP-glucose as sugar donor; Ka, kaempferol; Qu, quercetin; My, myricetin; Ap, apigenin; Lu, luteolin; Tri, tricetin; Na, naringenin; Gen, genistein; ECG, epicatechin gallate; (D) Representative HPLC chromatograph of the enzymatic products of the UGT73A17-1 protein at 30°C and 50°C, with quercetin as substrate and UDP-glucose as sugar donor; Q, quercetin; Q7G, quercetin 7-*O*-glucoside; (E) Comparison of the substrate conversion rate of the recombinant UGT73A17-1 protein at 30°C and 50°C, with nine flavonoids as substrates and UDP-glucose as sugar donor; Ka, kaempferol; Qu, quercetin; My, myricetin; Ap, apigenin; Lu, luteolin; Tri, tricetin; Na, naringenin; Gen, genistein; ECG, epicatechin gallate.

In the initial study of rUGT73A17 protein, we found it was capable of glucosylating epicatechin gallate as GbUGT716A1 [29], which is distinct from the other characterized UGTs. Therefore, we further identified the other flavonoid substrates of the recombinant UGT73A17 protein. It was surprising that it mainly catalyzes the formation of 7-*O*-glucosides (Fig 2). However, in another previous study, the recombinant UGT73A17-1 protein from a Japanese *Camellia sinensis* variety *sinensis* cultivar Yabukita, mainly forms 3-*O* glucosides with fewer flavonoid substrates [30], and they differ at only two amino acids (I296 and V466 for UGT73A17, and L296 and A466 for UGT73A17-1). Since the *UGT73A17-1* gene was not fully documented in the previous study [30], we therefore mutated these two residues to determine whether they were critical for the regio-specificity of UGT73A17 at either 3-*O* or 7-*O* position. Therefore, three site-directed mutagenesis of I296L, V466A and double substitution (I296L and V466A, designated as UGT73A17-1) were generated, and the activity of the corresponding recombinant proteins were analyzed and compared with that of UGT73A17 (Fig 6A, S2 Fig).

It showed that the recombinant proteins UGT73A17-I296L, UGT73A17-V466A, UGT73A17-1, produced mainly quercetin 7-*O*-glucosides with quercetin as substrate, which was the same as UGT73A17 (Fig 6B). In comparison with UGT73A17, the substrate conversion rates of UGT73A17-I296L, UGT73A17-V466A, UGT73A17-1 were lower toward all substrates except tricetin for UGT73A17-1 (Fig 6C).

To evaluate whether UGT73A17-1 was stable under heat treatment, the activity of rUGT73A17-1 protein was also analyzed at both 30°C and 50°C with quercetin as substrate. It showed that the product peak of UGT73A17-1 was significantly higher at 50°C than at 30°C, in particular Q7G (Fig 6D). In addition, the substrate conversion rate of UGT73A17-1 was higher toward all the six tested substrates at 50°C than at 30°C, in particular toward apigenin and luteolin (Fig 6E). This result further confirmed that both UGT73A17 and UGT73A17-1 are heat responsive protein in tea plant.

## Discussion

In this study, we identified *UGT73A17* gene from tea plant, which encodes a UGT protein that is active toward multiple flavonoid substrates, including flavonols (quercetin, kaempferol, and myricetin), flavones (apigenin, luteolin and tricetin), isoflavone (genistein), flavanone (naringenin) and epicatechin gallate (Fig 2). Tea plant possesses hundreds of *UGT* genes in the genome [21], but each *UGT* has distinct substrate profiles. For examples, we previously found that CsUGT78C14 and CsUGT78C14 were specifically responsible for the biosynthesis of flavonol 3-*O*-glucosides/galactosides, and CsUGT84A22 exhibited catalytic activity toward phenolic acids, in particular gallic acid for the production of the precursor of catechin gallates [21]. In another study, CsUGT73A20 was found to be active toward flavonols (kaempferol, quercetin, and myricetin), flavones (apigenin) and flavanone (naringenin) [31]. In comparison, CsUGT73A17 has a broader substrate profile than the other UGTs that were identified in tea plants so far. In particular, CsUGT73A17 also exhibited activity toward epicatechin gallate, which is the same as UGT716A1 from *Ginkgo biloba* that was active toward catechin galloylates [29], although the identity between them is only 30%. The glycosylated product of epigallocatechin gallate could increase water solubility, rapid metabolism and ready degradation in aqueous solutions, with significant bioactivity relating to human health [32–34]. Therefore, the property of epicatechin gallate produced by CsUGT73A17 could also be worth further investigation.

Both sequence and phylogenetic analyses demonstrated that CsUGT73A17 is closely related to 7-*O*-UGT (Fig 1). *In vitro* enzymatic assays also confirmed that CsUGT73A17 produced mainly 7-*O*-glucosides, with minor 3-, 3'-, 4'- *O*-glucosides (Fig 2). The enzymatic properties of CsUGT73A17 is distinct from CsUGT78A14 and CsUGT78A15 that produce only 3-*O*-glucoside/galactosides with regio-selectivity. CsUGT73A17 is similar to CsUGT73A20, which also produce mainly 7-*O*-glucosides, however, with minor 3-*O*-glucosides [31]. Considering the enzymatic kinetic properties, CsUGT73A17 showed high affinity with a lower *Km* values toward all flavonols and flavone substrate, but CsUGT73A20 appeared to be more active toward kaempferol than toward the other substrates (Fig 4).

In tea plant, flavonoids are mainly in form of 3-*O*-glucoside/galacosides [35], which may be produced mainly by CsUGT78A14 and CsUGT78A15 in immature leaves. However, neither CsUGT78A14 and CsUGT78A15 nor CsUGT73A20 are highly expressed in mature leaves, where rutin accumulated at a very high level [21]. Instead, UGT73A17 is highly expressed in mature leaves, which is possibly responsible for the production of rutin. Although CsUGT73A17 formed 7-*O*-glucosides as major products and 3-*O*-glucosides as minor products, it is still possible that it is responsible for the biosynthesis of rutin, since *in vitro* activity doesn't always reflect *in vivo* activity as for UGT in several previous studies [29, 31, 36, 37]. Taken together, these CsUGTs are most likely responsible for the accumulation of different flavonoid glucosides in different organs of tea plant.

Our study also demonstrated that CsUGT73A17 exhibited higher activity at relative high temperature (eg. 50°C), which is very unique among all CsUGTs that have been characterized in tea plant. Under heat condition, flavonoid glucosides/galactosides formed by CsUGT73A17 could be more effective to scavenge the free radical residues and reduce oxidation in the plant cells. In mature leaves, the inner environment might be more stressful than for young leaves, and the transcript level in mature leaves is higher than in other organs, implying CsUGT73A17 may play important role in the heat response in tea plant. In addition, multiple mono-, di- and tri-glucosides produced by CsUGT73A17 could diverse the flavonoid glucosides under stress.

Our study showed that UGT73A17 and UGT73A17-1 showed 99% identity, exhibited activity toward same substrates, possess identical kinetic parameters (Figs 4 and 5). In addition, they were both highly expressed in mature leaves and had the highest activity under heat condition (Fig 6) [30]. All these results implied that *UGT73A17* and *UGT73A17-1* are likely functional allelic genes in different tea plant germplasms with same function. Alletic variation in plants attributes in many aspects in plant development, yield, adaptability [38–41]. Therefore, *UGT73A17* and *UGT73A17-1* are likely to be functional alleles for flavonoid glycosylation in north Chinese and Japanese tea plants that grown in high latitude regions, respectively. Thus, the appearance of the alleles diversified flavonoid compounds, which could be powerful for plant survival under stress environment. It is also possible that *UGT73A17* and *UGT73A17-1* are functionally redundant genes in the expansion and evolution of *UGT* genes in tea plants, as in many other vascular plant lineages [42].

Our study demonstrated that CsUGT73A17 can produce multiple flavonoid glucosides, which could play important role in flavonoid profiles in tea, and further affect tea quality. Therefore, CsUGT73A17 is likely to be one of the key factors that affect tea quality, which have significant implication in the improvement of tea quality, in particular in mature leaves grown under high temperature regions.

## Conclusion

We isolated and characterized *UGT73A17* gene encodes UDP: flavonoid glucosyltransferase from tea plant. The recombinant CsUGT73A17 protein is active toward a broad range of flavonoid substrates, and it has a high activity at relatively high temperature for the production of multiple flavonoid glucosides. CsUGT73A17 is associated with tea quality and could be utilized in tea quality control.

### Accession numbers

The GenBank accession numbers and plant sources of the UGT protein sequences are: BvGT1, AAS94329 (*Beta vulgaris*); BvGT2, AAS94330 (*Beta vulgaris*); Cs3GT, AAS00612 (*Citrus sinensis*); CsUGT78A15, ALO19889 (*C*. *sinensis*); CsUGT78A14, ALO19888, (*C*. *sinensis*); UGT73A17, AB847095.1 (*C*. *sinensis*); DicGT1, BAD52003 (*Dianthus caryophyllus*); DicGT3, BAD52005 (*D*. *caryophyllus*); FaGT6, ABB92748 (*Fragaria × ananassa*); GeIF7GT, BAC78438 (*Glycyrrhiza echinata*); GhA5GT, BAA36423 (*Glandularia × hybrida*); Mt7GT, AAW56091 (*Medicago truncatula*); MtUGT71G1, AAW56092 (*M*. *truncatula*); NtUGT71A11, BAB88934 (*Nicotiana tabacum*); NtUGT71A6, BAB60720 (*N*. *tabacum*); NtUGT71A7, BAB60721 (*N*. *tabacum*); PfA5GT, BAA36421 (*Perilla frutescens var*. *crispa*); Ph3galT, AAD55985 (*Petunia × hybrida*); PhA5GT, BAA89009 (*P*. *hybrida*); RhGT4, BAE72453 (*Rosa hybrid*); Scb7GT, BAA83484 (*Scutellaria baicalensis*); SmGT, Q43641(*Solanum melongena*); ThA5GT, BAC54093 (*Torenia hybrid*); Vv3GT, AAB81682 (*Vitis vinifera*).

## Supporting information

S1 Table Primer sequences used in the present study. (PDF)

S2 Table Summary of the flavonoid substrates tested in this study. (PDF)

S1 Fig Multiple sequences alignment of the deduced CsUGT73A17 protein with other functionally characterized UGTs at amino acid level. Identical amino acids were indicated in black background, amino acids with 75% and 50% similarities were indicated in dark gray and the light grey, respectively. The GenBank accession numbers and plant sources of the respective protein sequences are: Scb7GT, BAA83484 (*Scutellaria baicalensis*); BvGT1, AAS94329 (*Beta vulgaris*); GeIF7GT, BAC78438 (*Glycyrrhiza echinata*) and Mt7GT, AAW56091 (*Medicago truncatula*).
(PDF)

S2 Fig The purified recombinant UGT proteins on 12% SDS-PAGE. From left to right: UGT73A17, UGT73A17-I296L, UGT73A17-V466A and UGT73A17-1, protein molecular marker.
(PDF)

S3 Fig Identification of the enzymatic products of the recombinant UGT73A17 protein toward quercetin as representative. From top to bottom: the enzymatic products of the recombinant UGT73A17 protein toward quercetin; authentic quercetin 7-*O*-glucoside (Q7G) standard; authentic quercetin 3-*O*-glucoside (Q3G) standards; Co-elution of authentic Q7G, Q3G and quercetin standards.
(PDF)

S4 Fig Mass spectra of the enzymatic products of recombinant UGT73A17 with epicatechin gallates as substrate. (PDF)

S5 Fig Analysis of the enzymatic products of the recombinant UGT73A17 protein toward apigenin and naringenin. (A-B) HPLC chromatograms of enzymatic product of the recombinant UGT73A17 protein with apigenin (A) and naringenin (B) as substrates at different temperature.
(PDF)

S6 Fig Analysis of the multiple glucoside products of the recombinant UGT73A17 protein with quercetin as substrate. (A) Reprehensive HPLC chromatography of the multiple quercetin glucosides formed by UGT73A17 at 50°C for 0.5 h (upper panel) and 2 h (lower panel). (B-J) Mass spectrum of the corresponding quercetin glycoside products labeled in (A).
(PDF)
