# Supplementary material for: Characterization of a heat responsive UDP: Flavonoid glucosyltransferase gene in tea plant (Camellia sinensis)
Source: PLoS One. 2018 Nov 26;13(11):e0207212. doi: 10.1371/journal.pone.0207212 (PMC6261043; doi:10.1371/journal.pone.0207212)
Supplement: S1 Table — (PDF) [file pone.0207212.s001.pdf]

S1 Table. Primer sequences used in the present study

**Primers**	**Sequence (5’-3’, the corresponding restriction sites were underlined)**	**Note**	**Restriction sites**
UGT73A17BF	CGGGATCCATGGCTAAGCTTCATTTCTTC	For gene cloning	*Bam* H Ⅰ
UGT73A17SR	ACGCGTCGACTTATGA ACTCATTTCTTGTATC	For gene cloning	*Sal* Ⅰ
UGT1-I296L-F	GGCGATTTTACTGCTTCTCAGTTGTTTGAGCTTGCGATGGG	For gene cloning	-
UGT1-I296L-R	GTTGCCCCGAAGCTTCAAGCCCCATCGCAAGCTCAAACAAC	For gene cloning	-
UGT1-V446A-F	CTGAGGGAATGAGAACCCGAGCTAGGGCAGCTAAGGATATG	For gene cloning	-
UGT1-V446A-R	TCTTCAACAGCCTTCTTCGCCATATCCTTAGCTGCCCTAGC	For gene cloning	-
UGT73A17RT-F	CACTTTGTGCGCTTCCGAGAA	For qRT-PCR	-
UGT73A17RT-R	GTCAGCTCCGTCTCTTTAACTT	For qRT-PCR	-
CHS1-RT-F	TGAAGGACCTGCCACGGTTATG	For qRT-PCR	-
CHS1-RT-R	GCCTTATGCTCGCTGTTTGT	For qRT-PCR	-
GAPDH-RT-F	TTGGCATCGTTGAGGGTCT	For qRT-PCR	
GAPDH-RT-R	CAGTGGGAACACGGAAAGC	For qRT-PCR	
AtRHM1-BF	CGGGATCCATGGCTTCGTACACTCCCAAGAAC	For gene cloning	*Bam H* Ⅰ
AtRHM1-XR	GCTCTAGATCAGGTTTTCTTGTTTGGCCCGTATG	For gene cloning	*Xba* Ⅰ
