# Supplementary material for: Characterization of a heat responsive UDP: Flavonoid glucosyltransferase gene in tea plant (Camellia sinensis)
Source: PLoS One. 2018 Nov 26;13(11):e0207212. doi: 10.1371/journal.pone.0207212 (PMC6261043; doi:10.1371/journal.pone.0207212)
Supplement: S2 Table — (PDF) [file pone.0207212.s002.pdf]

S2 Table. Summary of the flavonoid substrates tested in this study

Classes	Substrates	Abbreviations
Flavonols	kaempferol	Ka
	quercetin	Qu
	myricetin	My
Flavones	apigenin	Ap
	luteolin	Lu
	tricetin	Tri
Flavanone	naringenin	Na
Isoflavones	daidzein	Da
	genistein	Gen
Non-galloylated catechins	catechin	C
	epicatechin	EC
	gallocatechin	GC
	epigallocatechin	EGC
Galloylated catechins	catechin gallate	CG
	gallocatechin gallate	GCG
	epicatechin gallate	ECG
	epigallocatechin gallate	EGCG
