# Supplementary material for: Characterization of a heat responsive UDP: Flavonoid glucosyltransferase gene in tea plant (Camellia sinensis)
Source: PLoS One. 2018 Nov 26;13(11):e0207212. doi: 10.1371/journal.pone.0207212 (PMC6261043; doi:10.1371/journal.pone.0207212)
Supplement: S1 Fig — Identical amino acids were indicated in black background, amino acids with 75% and 50% similarities were indicated in dark gray and the light grey, respectively. The GenBank accession numbers and plant sources of the respective protein sequences are: Scb7GT, BAA83484 (Scutellaria baicalensis); BvGT1, AAS94329 (Beta vulgaris); GeIF7GT, BAC78438 (Glycyrrhiza echinata) and Mt7GT, AAW56091 (Medicago truncatula). (PDF) [file pone.0207212.s003.pdf]

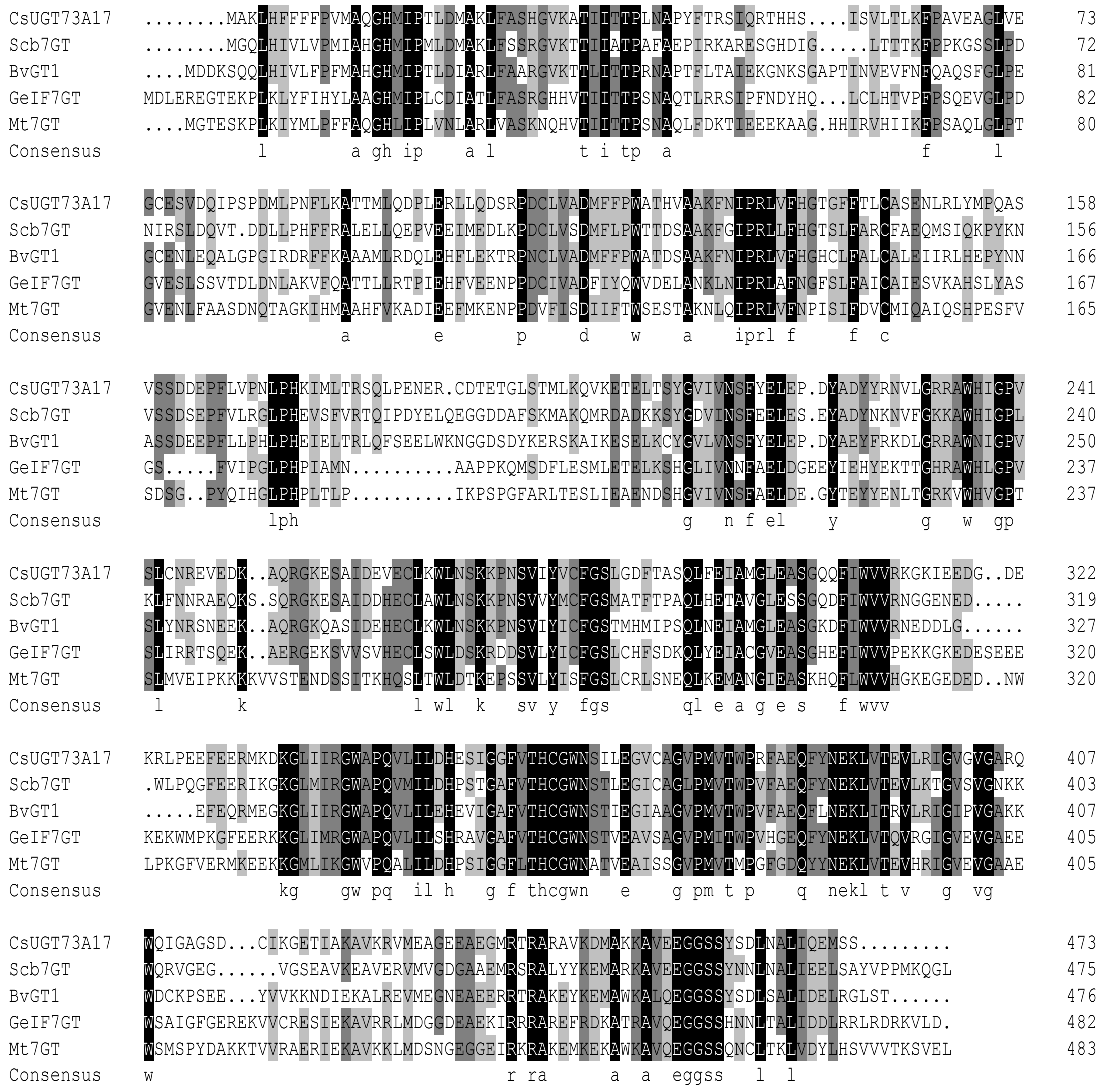

S1 Fig. Multiple sequences alignment of the deduced CsUGT73A17 protein with other functionally characterized UGTs at amino acid level. Identical amino acids were indicated in black background, amino acids with 75% and 50% similarities were indicated in dark gray and the light grey, respectively. The GenBank accession numbers and plant sources of the respective protein sequences are: Scb7GT, BAA83484 (*Scutellaria baicalensis*); BvGT1, AAS94329 (*Beta vulgaris*); GeIF7GT, BAC78438 (*Glycyrrhiza echinata*) and Mt7GT, AAW56091 (*Medicago truncatula*).
