# Supplementary material for: Characterization of a heat responsive UDP: Flavonoid glucosyltransferase gene in tea plant (Camellia sinensis)
Source: PLoS One. 2018 Nov 26;13(11):e0207212. doi: 10.1371/journal.pone.0207212 (PMC6261043; doi:10.1371/journal.pone.0207212)
Supplement: S2 Fig — From left to right: UGT73A17, UGT73A17-I296L, UGT73A17-V466A and UGT73A17-1, protein molecular marker. (PDF) [file pone.0207212.s004.pdf]

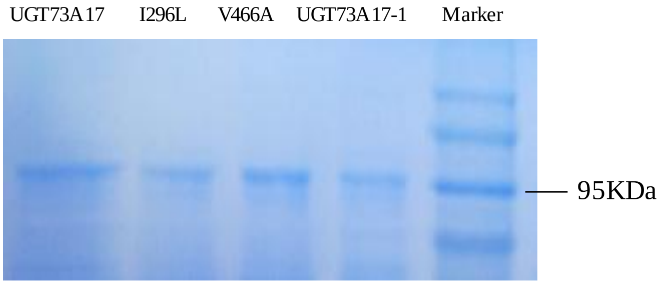

S2 Fig. The purified recombinant UGT proteins on 12% SDS-PAGE. From left to right: UGT73A17, UGT73A17-I296L, UGT73A17-V466A and UGT73A17-1, protein molecular marker.
