# Supplementary material for: Characterization of a heat responsive UDP: Flavonoid glucosyltransferase gene in tea plant (Camellia sinensis)
Source: PLoS One. 2018 Nov 26;13(11):e0207212. doi: 10.1371/journal.pone.0207212 (PMC6261043; doi:10.1371/journal.pone.0207212)
Supplement: S3 Fig — From top to bottom: the enzymatic products of the recombinant UGT73A17 protein toward quercetin; authentic quercetin 7-O-glucoside (Q7G) standard; authentic quercetin 3-O-glucoside (Q3G) standards; Co-elution of authentic Q7G, Q3G and quercetin standards. (PDF) [file pone.0207212.s005.pdf]

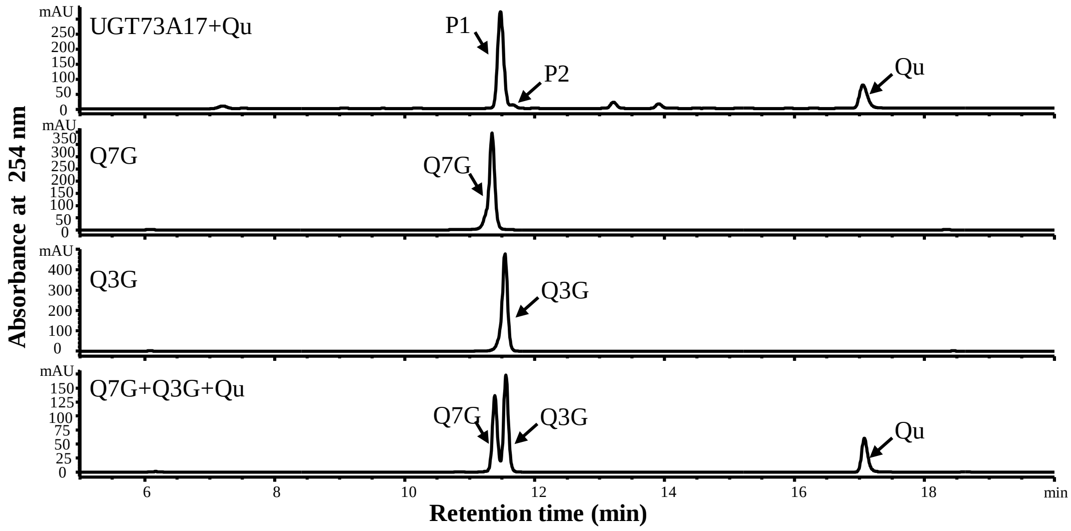

S3 Fig. Identification of the enzymatic products of the recombinant UGT73A17 protein toward quercetin as representative. From top to bottom: the enzymatic products of the recombinant UGT73A17 protein toward quercetin; authentic quercetin 7-*O*-glucoside (Q7G) standard; authentic quercetin 3-*O*-glucoside (Q3G) standards; Co-elution of authentic Q7G, Q3G and quercetin standards.
