# Supplementary material for: Characterization of a heat responsive UDP: Flavonoid glucosyltransferase gene in tea plant (Camellia sinensis)
Source: PLoS One. 2018 Nov 26;13(11):e0207212. doi: 10.1371/journal.pone.0207212 (PMC6261043; doi:10.1371/journal.pone.0207212)
Supplement: S4 Fig — (PDF) [file pone.0207212.s006.pdf]

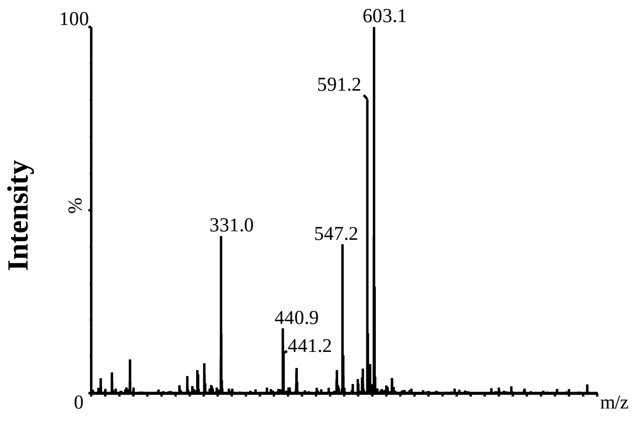

S4 Fig. Mass spectra of the enzymatic products of recombinant UGT73A17 with epicatechin gallate as substrate.
