# Supplementary material for: Characterization of a heat responsive UDP: Flavonoid glucosyltransferase gene in tea plant (Camellia sinensis)
Source: PLoS One. 2018 Nov 26;13(11):e0207212. doi: 10.1371/journal.pone.0207212 (PMC6261043; doi:10.1371/journal.pone.0207212)
Supplement: S5 Fig — (A-B) HPLC chromatograms of enzymatic product of the recombinant UGT73A17 protein with apigenin (A) and naringenin (B) as substrates at different temperature. (PDF) [file pone.0207212.s007.pdf]

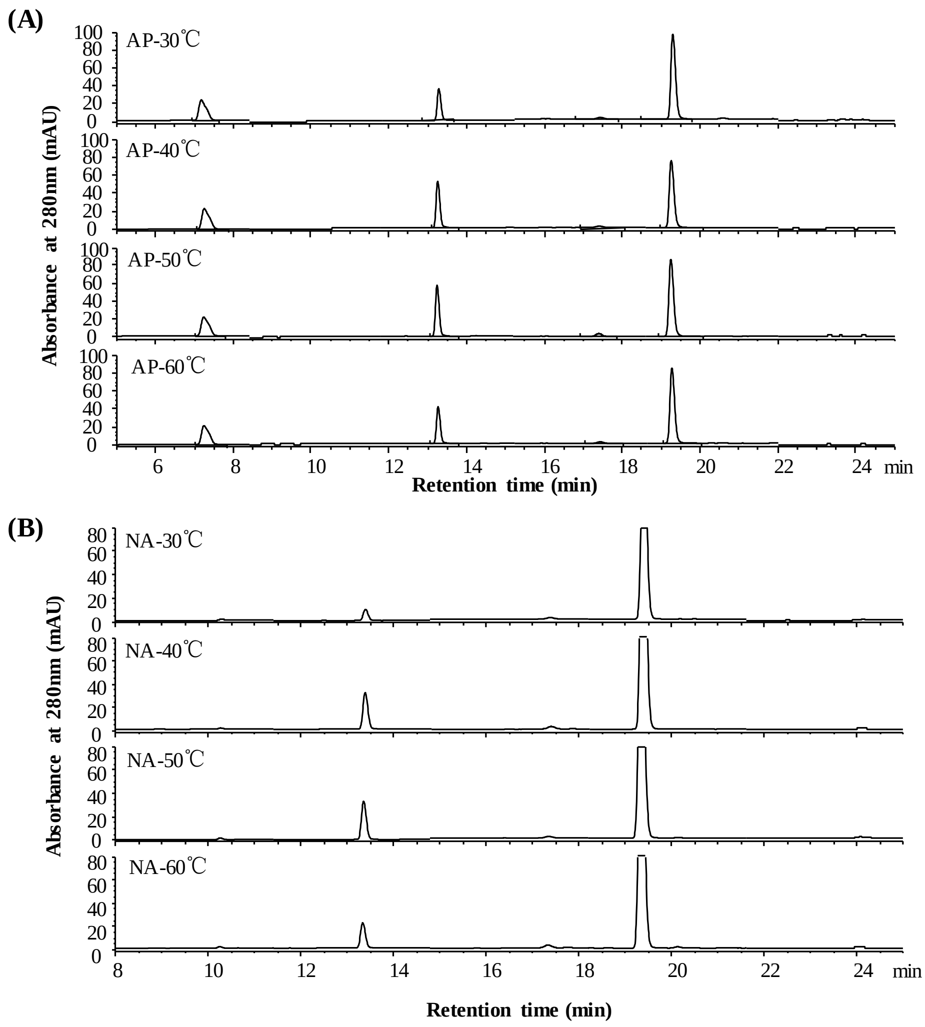

S5 Fig. Analysis of the enzymatic products of the recombinant UGT73A17 protein toward apigenin and naringenin. (A-B) HPLC chromatograms of enzymatic product of the recombinant UGT73A17 protein with apigenin (A) and naringenin (B) as substrates at different temperature.
