# Supplementary material for: Characterization of a heat responsive UDP: Flavonoid glucosyltransferase gene in tea plant (Camellia sinensis)
Source: PLoS One. 2018 Nov 26;13(11):e0207212. doi: 10.1371/journal.pone.0207212 (PMC6261043; doi:10.1371/journal.pone.0207212)
Supplement: S6 Fig — (A) Reprehensive HPLC chromatography of the multiple quercetin glucosides formed by UGT73A17 at 50°C for 0.5 h (upper panel) and 2 h (lower panel). (B-J) Mass spectrum of the corresponding quercetin glycoside products labeled in (A). (PDF) [file pone.0207212.s008.pdf]

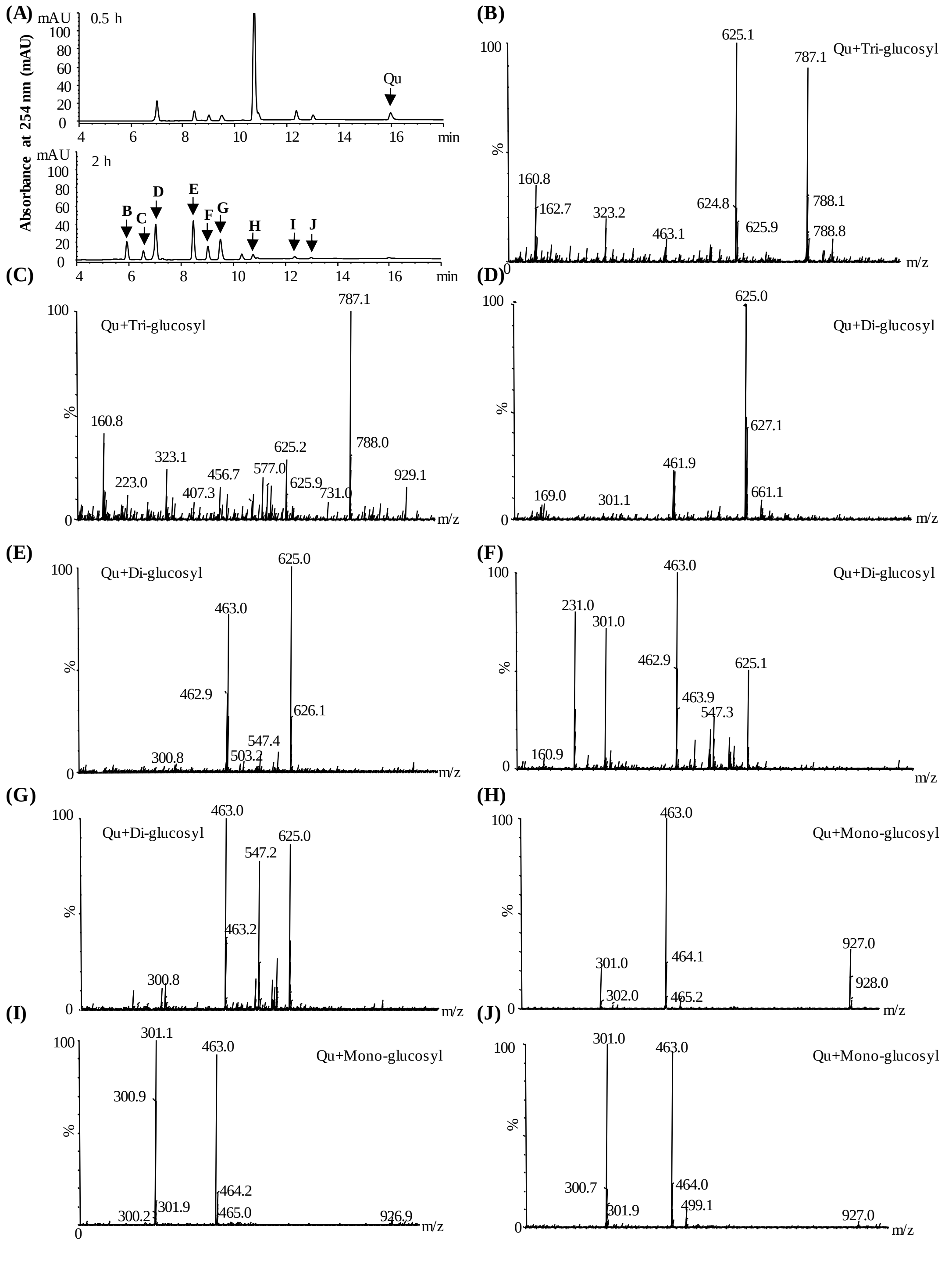

S6 Fig. Analysis of the multiple glucoside products of the recombinant UGT73A17 protein with quercetin as substrate. (A) Reprehensive HPLC chromatography of the multiple quercetin glucosides formed by UGT73A17 at 50℃ for 0.5 h (upper panel) and 2 h (lower panel). (B-J) Mass spectrum of the corresponding quercetin glycoside products labeled in (A).
